# Postoperative vision loss due to bilateral vitreous hemorrhage after robot-assisted laparoscopic hysterectomy: A case report

**DOI:** 10.1016/j.ajoc.2023.101985

**Published:** 2023-12-26

**Authors:** Yuka Numaga, Fumiyuki Araki, Kunihiro Azuma, Taku Toyama, Koichiro Sugimoto, Tomoyasu Shiraya, Takashi Ueta

**Affiliations:** a Department of Ophthalmology, Graduate School of Medicine and Faculty of Medicine, The University of Tokyo, 7-3-1, Hongo, Bunkyo, Tokyo, 113-8655, Japan; b Eye Center, Showa General Hospital, 8-1-1 Hanakoganei, Kodaira, Tokyo, 187-8510, Japan

**Keywords:** Robot-assisted surgery, Postoperative vision loss, Vitreous hemorrhage, Preretinal hemorrhage, Trendelenburg position, Peumoperitoneum

## Abstract

**Purpose:**

To report a case of bilateral vitreous hemorrhage (VH) resulting in postoperative vision loss (POVL) after robot-assisted laparoscopic hysterectomy in a 71-year-old female patient.

**Observations:**

At initial presentation, best-corrected visual acuity was hand motion at 20 cm in the right eye and 20/666 in the left eye. VH in both eyes and preretinal hemorrhage in the left eye was observed. As the hemorrhage gradually resolved, a full-thickness macular hole was discovered in the right eye, for which the patient did not agree with a surgical treatment.

**Conclusions and importance:**

This report describes a rare incidence of bilateral VH as a cause of POVL after non-ophthalmic surgery, which may be related to Trendelenburg positioning, CO2 pneumoperitoneum, and a long surgical duration. Given that POVL can cause severe visual impairment, consultation with ophthalmologists is crucial.

## 1 Introduction

Robot-assisted surgery has dramatically expanded its clinical applications, both in terms of surgical volume and the types of surgeries performed, since receiving approval from the Food and Drug Administration in 2005. Hysterectomy for gynecologic cancers is currently one of the major procedures performed through robotic assistance.^1^ In Japan, the number of robot-assisted laparoscopic hysterectomies (RALHs) has been increasing since it became covered by public insurance in 2018.^2^

Compared to conventional laparoscopic surgery, RALH has several advantages, including fewer complications, fewer hospital stays, less blood loss, lower likelihood of transitioning to laparotomy during surgeries. However, because patients are placed in a steep Trendelenburg position with CO_2_ pneumoperitoneum, studies have reported a risk of increased intraocular pressure and ischemic optic neuropathy.^3^ On the other hand, vitreous hemorrhage (VH) as an etiology of post-operative vision loss (POVL) associated with RALH or non-ophthalmic surgeries has not been understood.

## 2 Case report

A 71-year-old female patient without a hemocoagulability issue underwent RALH, bilateral salpingectomy, and pelvic lymphadenectomy after being diagnosed with stage IB endometrial cancer. She had a history of normal tension glaucoma and pathologic myopia but no prior ophthalmic surgeries. The most recent eye examination before the RALH occurred four months earlier at a local ophthalmic clinic. During that examination, the best-corrected visual acuity (BCVA) was recorded as 20/50 in the right eye with −20.0D myopia and 20/20 in the left eye with −13.5D myopia. The relatively diminished BCVA in the right eye had remained stable, and was attributed to diffuse chorioretinal atrophy associated with pathologic myopia. The operation of RALH lasted 4 hours and 58 minutes with minimal bleeding, and Trendelenburg positioning was used during the surgery with the patient's head positioned downward at 20° instead of the usual 25° due to her history of glaucoma. However, immediately after the surgery, the patient experienced visual impairment in both eyes.

**Fig. 1:**
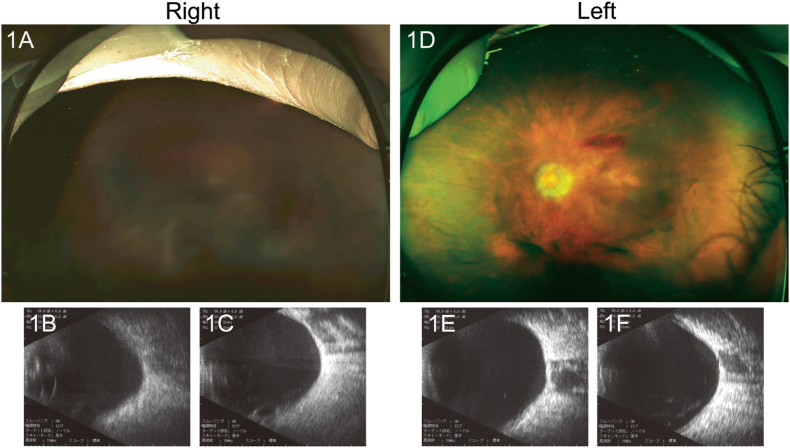
Fundus photographs (1A and 1D) and B-mode images in horizontal (1B and 1E) and in sagittal (1C and 1F) sections at presentation. Dense vitreous hemorrhage without retinal detachment was observed in the right eye. Mild vitreous and preretinal hemorrhage was observed in the left eye.

Upon presentation to our ophthalmic service, the patient's best-corrected visual acuity (BCVA) was hand motion at 20 cm in her right eye and 20/666 Snellen equivalent in her left eye. Intraocular pressure was 15 mmHg in the right eye and 17 mmHg in the left eye. The fundus in her right eye was invisible due to massive VH, and B-scan ultrasound ruled out retinal detachment (Fig. 1). In her left eye, VH and preretinal hemorrhage were observed (Fig. 1).

**Fig. 2:**
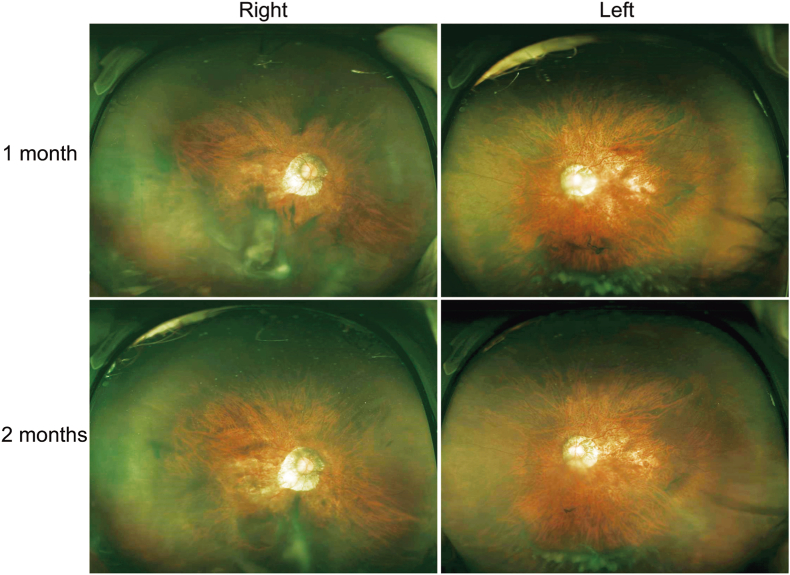
Fundus photographs One and two months after the initial presentation. Vitreous and preretinal hemorrhage subsided gradually.

**Fig. 3:**
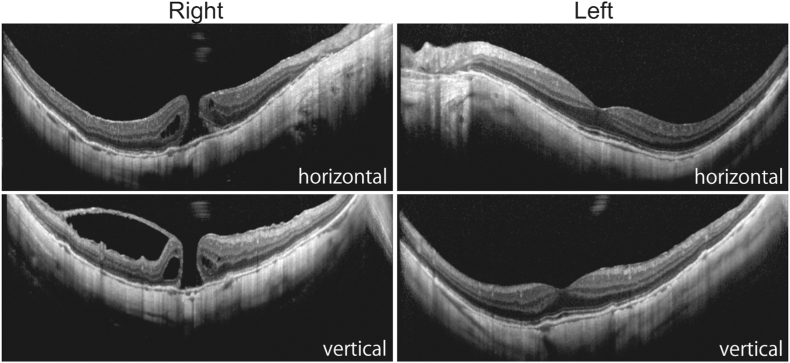
Spectral-domain optical coherence tomography two months after the initial presentation. A full-thickness macular hole was observed in the right eye.

**Fig. 4:**
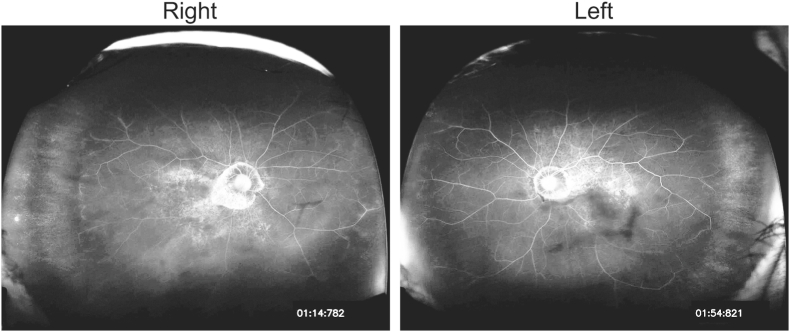
Fluorescein angiography seven months after the initial presentation. Retinal vascular abnormalities including neovascularization, nonperfusion area were not found. Hyperfluorescence at posterior pole is window defects due to diffuse chorioretinal atrophy related to myopic maculopathy.

After careful observation over the course of two months, the VH and preretinal hemorrhage gradually resolved, and the patient's BCVA improved to 20/2000 in the right eye and 20/40 in the left eye (Fig. 2). However, spectral domain-optical coherence tomography revealed the presence of a full-thickness macular hole in the patient's right eye (Fig. 3). Although vitrectomy was recommended to treat the macular hole, the patient declined the procedure due to the severe malaise she experienced following therapies for endometrial cancer. Fluorescent angiography was conducted five months after the initial presentation, which did not reveal any non-perfusion areas or neovascularization in either eye (Fig. 4).

## 3 Discussion

POVL after non-ophthalmic surgery is a rare complication, with reported incidence rates ranging from 0.013 to 0.2 %.^4^ Common causes of POVL include ischemic optic neuropathy, central retinal artery occlusion, and cortical blindness, and the risk of POVL is considered to be higher in patients undergoing surgery in the Trendelenburg position, as well as those who experience hypoperfusion during general anesthesia.^4^ During robot-assisted laparoscopic surgery, the Trendelenburg position is often required for extended periods, and CO_2_ pneumoperitoneum can further increase arterial and venous pressure in the eye. Indeed, previous studies have reported an association between robot-assisted laparoscopic surgery and increased intraocular pressure and ischemic optic neuropathy.^5^, ^6^, ^7^, ^8^ While the Trendelenburg position is crucial for enhancing surgical access to pelvic organs by repositioning abdominal organs toward the head, modifications have been explored in order to mitigate potential complications. An example is positioning patients with their head and shoulders maintained horizontally,^9^ or by decreasing the degree of steepness, as applied in our case. To the best of our knowledge, VH has not been reported as a cause of POVL following surgeries involving Trendelenburg position.

We speculated that the vitreous and preretinal hemorrhage in this case could be attributed to one or several of the following potential causes: 1) increased arterial and venous pressure resulting from the extended surgical time in Trendelenburg position and pneumoperitoneum, 2) retinal fragility due to high myopia with posterior staphyloma and advanced age, 3) Valsalva hemorrhagic retinopathy caused by coughing associated with tracheal irritation during and after extubation.^10^ Etiological similarity is observed in the several reported cases of bilateral bleeding in the ear associated with Trendelenburg position and pneumoperitoneum during laparoscopic surgery.^11^^,^^12^ The authors of these reports have also discussed the potential causal effect of Trendelenburg position with pneumoperitoneum, leading to increased arterial and venous pressure, and hemorrhage from the capillaries.

The etiology of macular hole development in this case remains uncertain. Even though the BCVA was confirmed to be stable in both eyes four months prior to RALH, it is still conceivable that the MH existed before RALH but was concealed by the presence of VH.

In conclusion, we report a rare case of VH as an etiology of POVL after robot-assisted laparoscopic surgery. It is important to consider the potential risk of POVL in patients undergoing laparoscopic surgeries, particularly with prolonged Trendelenburg positioning and pneumoperitoneum. Consultation with ophthalmologists is important, as the prognosis of POVL after non-ophthalmic surgeries is not always favorable.

## Funding

This work was supported by 10.13039/501100001691JSPS KAKENHI Grant Number 21K09758 to Takashi Ueta, 22K16963 to Taku Toyama, and 20K18377 to Fumiyuki Araki.

Consent to publish the case report was not obtained. This report does not contain any personal information that could result in patient identification.

All authors attest that they meet the current ICMJE criteria for Authorship.

## CRediT authorship contribution statement

**Yuka Numaga:** Data curation, Formal analysis, Investigation, Writing – original draft. **Fumiyuki Araki:** Conceptualization, Funding acquisition, Investigation, Methodology, Resources, Supervision, Writing – review & editing. **Kunihiro Azuma:** Data curation, Investigation, Methodology, Supervision, Writing – review & editing. **Taku Toyama:** Conceptualization, Methodology, Validation, Writing – original draft. **Koichiro Sugimoto:** Methodology, Supervision, Validation, Writing – review & editing. **Tomoyasu Shiraya:** Conceptualization, Funding acquisition, Supervision, Validation, Writing – review & editing. **Takashi Ueta:** Conceptualization, Formal analysis, Funding acquisition, Methodology, Validation, Writing – review & editing.

## Declaration of competing interest

The authors declare that they have no known competing financial interests or personal relationships that could have appeared to influence the work reported in this paper.
